# Lifting lockdown COVID19 restrictions: What can pharmacists do as the world wakes up?

**DOI:** 10.1016/j.rcsop.2021.100028

**Published:** 2021-06-12

**Authors:** Fatemah Ashkanani, Rebecca Richardson, Laura Lindsey, Adam Pattison Rathbone

**Affiliations:** a School of Pharmacy, Newcastle University, Newcastle upon Tyne, UK; b Sunderland GP Alliance Medical Practices, Sunderland, UK

In January 2020, the WHO Emergency Committee declared a global health emergency due to growing cases of the novel coronavirus infection (COVID-19).^1^ Countries worldwide announced lockdowns to contain the virus and prevent its spread, within schools, workplaces, and homes.^2^ The impact of the pandemic on individuals is multifaceted. Indeterminate predictions about the future, conflicting information and obligations to unfamiliar health procedures that interfered with personal freedoms caused concerns and emotional distress.^3^ With the increasing number of COVID-19 cases and deaths, people began to feel stressed, worried, and anxious. Many people experienced isolation, adapted to wearing face-covering, adhered to social distancing, and had limited access to recreational spaces, such as gyms, which exacerbated stress, anxiety, and depression – as well as sleep disturbances.^1^

During the pandemic, the focus of health care providers was on (and is still on) the virus; however, this may have reduced attention on other conditions, such as sleep disturbances. As practitioners, it is important to remember the bi-directional relationship between poor sleep and poor mental health and engagement in social life. Sleep disturbance can disrupt pro-social behaviours and is a risk factor for suicidal ideation, suicidal behaviours, and death.^4^ Recognizing and treating sleep disorders is important for both people with and without existing physical and mental health conditions.^4^

Although social restrictions may be considered to have ‘freed up time’ to provide opportunities to take a break from the ‘hustle and bustle’ of everyday 21st century life,^5^ limited access to recreational and therapeutic spaces may have disrupted the natural sleep-wake cycles for many. In addition, people were advised not to attend care settings unnecessarily,^6^ so minor ailments, such as sleep disturbances, may have gone untreated. However, prolonged lockdowns may have led some people to adapt their sleeping habits, to align with their new socially distanced life.^7^ As the UK moves out of lockdown then, people may be overwhelmed by the dramatic change in their everyday lives, forced again to adapt their sleeping habits to meet pre-COVID19 routines. Pharmacists then may have a key role ahead, identifying sleep disturbances and providing recommendations for therapeutic approaches to re-establish sleeping rhythms.

There is evidence exploring pharmacists' roles in supporting sleep.^7^, ^8^, ^9^, ^10^, ^11^ Research has shown that pharmacists can identify, screen, and manage sleep conditions as autonomous practitioners. There is an opportunity here to help people with existing sleep disorders developing during lockdown or identifying those at risk of developing it during the easing of restrictions. Alongside dispensing prescribed medications and over-the-counter sleeping aids, pharmacists can provide non-pharmacological treatments for those with sleep difficulties. For example, advising people about sleep hygiene, recommending, or even providing cognitive behavioural therapy for insomnia (CBT—I). CBT-I is the first line therapy for chronic insomnia^12^, ^13^, ^14^ and evidence suggests that it can be provided by non-sleep specialists.^15^, ^16^, ^17^, ^18^, ^19^ It is an under researched area but evidence has demonstrated that pharmacists can effectively deliver CBT-I to people with chronic insomnia.^20^ Trained community pharmacists have successfully delivered behavioural treatments to people with insomnia.^21^ The easing of restrictions then presents a new opportunity for pharmacists to support people to sleep well, as the world around us ‘wakes up’.

Fatemah Ashkanani is a PhD Candidate and Kuwaiti-registered Pharmacist.

Rebecca Richardson is a General Practice Pharmacist in Sunderland.

Laura Lindsey is a Lecturer in Pharmacy Practice.

Adam Pattison Rathbone is Lecturer in Clinical and Social Pharmacy.

## Funding

This study was completed as part of doctoral studies funded by 10.13039/501100004726Ministry of Health, Kuwait and 10.13039/501100000774Newcastle University, United Kingdom.

## Declaration of Competing Interest

The authors declare that they have no known competing financial interests or personal relationships that could have appeared to influence the work reported in this paper.
